# *N*,*N*′-Bis(3-methyl­phen­yl)propane­diamide

**DOI:** 10.1107/S1600536810044089

**Published:** 2010-10-31

**Authors:** B. Thimme Gowda, Miroslav Tokarčík, Vinola Z. Rodrigues, Jozef Kožíšek, Hartmut Fuess

**Affiliations:** a Department of Chemistry, Mangalore University, Mangalagangotri 574 199, Mangalore, India; b Faculty of Chemical and Food Technology, Slovak Technical University, Radlinského 9, SK-812 37 Bratislava, Slovak Republic; c Institute of Materials Science, Darmstadt University of Technology, Petersenstrasse 23, D-64287 Darmstadt, Germany

## Abstract

The mol­ecular structure of the title compound, C_17_H_18_N_2_O_2_, is symmetrical around the central C atom. The two halves of the mol­ecule are related by a twofold rotation axis. In each half of the mol­ecule, the structure is stabilized by intra­molecular C—H⋯O hydrogen bonds. Furthermore, each amide group is almost coplanar with the adjacent benzene ring [dihedral angle is 9.2 (2)°]. The planes of the amide groups are inclined at an angle of 68.5 (1)°, while the two benzene rings make a dihedral angle of 70.40 (3)°. In the crystal, mol­ecules are linked by inter­molecular N—H⋯O hydrogen bonds into chains running along the *c* axis. Neighbouring chains are weakly coupled by π–π stacking inter­actions [centroid–centroid distance = 3.7952 (8) Å].

## Related literature

For related compounds, see: Gowda *et al.* (2007 ▶, 2009 ▶, 2010 ▶).
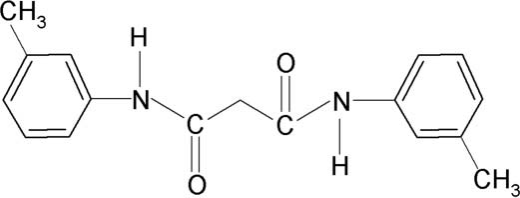


## Experimental

### 

#### Crystal data

C_17_H_18_N_2_O_2_
*M* *_r_* = 282.33
Monoclinic, 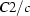

*a* = 15.3617 (6) Å
*b* = 11.2277 (6) Å
*c* = 8.7316 (5) Å
β = 90.370 (4)°
*V* = 1505.97 (13) Å^3^
*Z* = 4
Mo *K*α radiation
μ = 0.08 mm^−1^
*T* = 295 K
0.58 × 0.27 × 0.16 mm

#### Data collection

Oxford Diffraction Gemini R CCD diffractometer
Absorption correction: multi-scan (*CrysAlis PRO*; Oxford Diffraction, 2009 ▶) *T*_min_ = 0.970, *T*_max_ = 0.989
11578 measured reflections
1457 independent reflections
1252 reflections with *I* > 2σ(*I*)
*R*_int_ = 0.019

#### Refinement

*R*[*F* ^2^ > 2σ(*F* ^2^)] = 0.032
*wR*(*F* ^2^) = 0.096
*S* = 1.05
1457 reflections
101 parameters
1 restraint
H atoms treated by a mixture of independent and constrained refinement
Δρ_max_ = 0.15 e Å^−3^
Δρ_min_ = −0.13 e Å^−3^

### 

Data collection: *CrysAlis PRO* (Oxford Diffraction, 2009 ▶); cell refinement: *CrysAlis PRO*; data reduction: *CrysAlis PRO*; program(s) used to solve structure: *SHELXS97* (Sheldrick, 2008 ▶); program(s) used to refine structure: *SHELXL97* (Sheldrick, 2008 ▶); molecular graphics: *ORTEP-3* (Farrugia, 1997 ▶) and *DIAMOND* (Brandenburg, 2002 ▶); software used to prepare material for publication: *SHELXL97*, *PLATON* (Spek, 2009 ▶) and *WinGX* (Farrugia, 1999 ▶).

## Supplementary Material

Crystal structure: contains datablocks I, global. DOI: 10.1107/S1600536810044089/bq2245sup1.cif

Structure factors: contains datablocks I. DOI: 10.1107/S1600536810044089/bq2245Isup2.hkl

Additional supplementary materials:  crystallographic information; 3D view; checkCIF report

## Figures and Tables

**Table 1:** Hydrogen-bond geometry (Å, °)

*D*—H⋯*A*	*D*—H	H⋯*A*	*D*⋯*A*	*D*—H⋯*A*
C6—H6⋯O1	0.93	2.34	2.9124 (14)	120
N1—H1*N*⋯O1^i^	0.86	2.16	2.9932 (12)	162
C8—H8⋯O1^i^	0.97 (1)	2.54 (1)	3.3981 (9)	149 (1)

Symmetry code: (i) 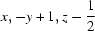.

**Fig. 1.**
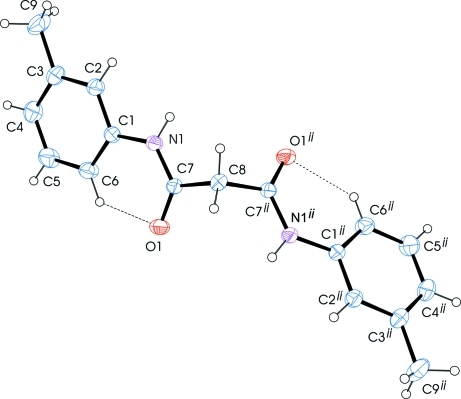
Molecular structure of (I) showing the atom labeling scheme. Displacement ellipsoids are drawn at the 30% probability level. The molecule consists of two identical parts related by the symmetry operator (ii): -x + 1, y, -z + 1/2 with C8 atom as the center. Two intramolecular hydrogen bonds are shown as dashed lines.

**Fig. 2.**
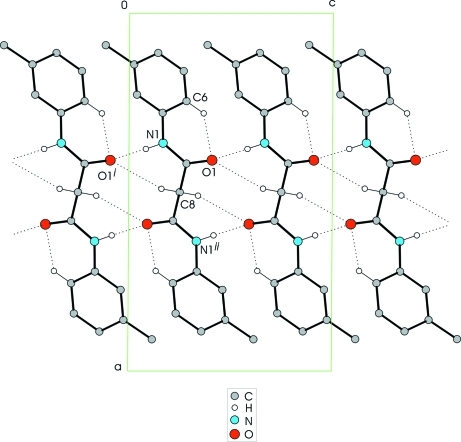
Crystal structure of (I) showing infinite molecular chain running along the c axis, generated by intermolecular N—H···O(i) hydrogen bonds shown as dashed lines. H atoms not participating in hydrogen bonding have been omitted. Symmetry operators (i): x, -y + 1, z - 1/2; (ii): -x + 1, y, -z + 1/2.

## supplementary crystallographic information

### Comment

The amide moiety is an important constituent of many biologically important compounds. As a part of studying the substituent effects on the structures of this class of compounds (Gowda *et al.*, 2007; 2009; 2010), the crystal structure of *N*,*N*-bis(3-methylphenyl)- propanediamide has been determined (I) (Fig. 1).

The molecule of (I) is symmetrical around the central carbon atom C8. The two halves of the molecule are related by the symmetry (-*x* + 1,*y*,-*z* + 1/2), which is a twofold rotation axis. The molecular structure is stabilized by the C–H···O intramolecular hydrogen bond in each half of the molecule (Table 1). In the geometry of the molecule, each amide group is almost coplanar with the adjacent phenyl ring, as indicated by the dihedral angle of 9.2 (2)°. The planes of amide groups are inclined at an angle of 68.5 (1)°, while the two phenyl rings make a dihedral angle of 70.40 (3)°. In the crystal, the molecules are linked by intermolecular N–H···O hydrogen bonds into the chains running along the *c* axis (Fig. 2). The neighboring chains are weakly coupled by π–π stacking interaction between the phenyl ring centroid *Cg*1 at the position (*x*,*y*,*z*) and the centroid *Cg*1 at the position (1/2 - *x*,1/2 - *y*,-*z*). The stacking geometry is such that the interplanar distance of the rings is 3.5290 (5) Å, the centroid-centroid distance is 3.7952 (8)Å and the offset is 1.396 (1) Å.

### Experimental

Malonic acid (0.3 mol) in dichloromethane (30 ml) was treated with *m*-toluidine (0.6 mol) in dichloromethane (30 ml), dropwise with stirring. The resulting mixture was stirred for 3 hrs and kept aside for 12 hrs for the completion of reaction and evaporation of the solvent, dichloromethane. The product obtained was added to crushed ice to obtain the precipitate. The latter was thoroughly washed with water and then with saturated sodium bicarbonate solution and washed again with water. It was then given a wash with 2 N HCl. It was again washed with water, filtered, dried and recrystallized to the constant melting point from ethanol.

Prism like colorless single crystals of the title compound used in X-ray diffraction studies were obtained by a slow evaporation of its ehanolic solution at room temperature.

### Refinement

All hydrogen atoms, except for H atoms attached to C8, were positioned geometrically and refined using a riding model with C–H = 0.93 or 0.96 Å and N–H = 0.86 Å. The *U*_iso_(H) values were set at 1.2*U*_eq_(C, N) or 1.5*U*_eq_(*C*-methyl). The hydrogen atom attached to the central C8 atom was refined freely with the bond length restrained to 0.93 (3) Å. The second hydrogen atom attached to C8 is positioned *via* the symmetry operator (ii): -*x* + 1,*y*,-*z* + 1/2. The C9-methyl group was treated as orientational disordered in the positions of H atoms. Two sets of methyl hydrogen atoms were refined with equal occupancies of 0.50.

### Crystal data

**Table:**

C_17_H_18_N_2_O_2_	*F*(000) = 600
*M**_r_* = 282.33	*D*_x_ = 1.245 Mg m^−^^3^
Monoclinic, *C*2/*c*	Mo *K*α radiation, λ = 0.71073 Å
Hall symbol: -C 2yc	Cell parameters from 7458 reflections
*a* = 15.3617 (6) Å	θ = 3.5–29.5°
*b* = 11.2277 (6) Å	µ = 0.08 mm^−^^1^
*c* = 8.7316 (5) Å	*T* = 295 K
β = 90.370 (4)°	Prism, colorless
*V* = 1505.97 (13) Å^3^	0.58 × 0.27 × 0.16 mm
*Z* = 4	

### Data collection

**Table:**

Oxford Diffraction Gemini R CCD diffractometer	1457 independent reflections
graphite	1252 reflections with *I* > 2σ(*I*)
Detector resolution: 10.434 pixels mm^-1^	*R*_int_ = 0.019
ω scans	θ_max_ = 25.8°, θ_min_ = 3.2°
Absorption correction: multi-scan (*CrysAlis PRO*; Oxford Diffraction, 2009)	*h* = −18→18
*T*_min_ = 0.970, *T*_max_ = 0.989	*k* = −13→13
11578 measured reflections	*l* = −10→10

### Refinement

**Table:**

Refinement on *F*^2^	Secondary atom site location: difference Fourier map
Least-squares matrix: full	Hydrogen site location: inferred from neighbouring sites
*R*[*F*^2^ > 2σ(*F*^2^)] = 0.032	H atoms treated by a mixture of independent and constrained refinement
*wR*(*F*^2^) = 0.096	*w* = 1/[σ^2^(*F*_o_^2^) + (0.0517*P*)^2^ + 0.487*P*] where *P* = (*F*_o_^2^ + 2*F*_c_^2^)/3
*S* = 1.05	(Δ/σ)_max_ < 0.001
1457 reflections	Δρ_max_ = 0.15 e Å^−^^3^
101 parameters	Δρ_min_ = −0.13 e Å^−^^3^
1 restraint	Extinction correction: *SHELXL97* (Sheldrick, 2008), Fc^*^=kFc[1+0.001xFc^2^λ^3^/sin(2θ)]^-1/4^
Primary atom site location: structure-invariant direct methods	Extinction coefficient: 0.017 (2)

### Special details

**Table:**

Geometry. All e.s.d.'s (except the e.s.d. in the dihedral angle between two l.s. planes) are estimated using the full covariance matrix. The cell e.s.d.'s are taken into account individually in the estimation of e.s.d.'s in distances, angles and torsion angles; correlations between e.s.d.'s in cell parameters are only used when they are defined by crystal symmetry. An approximate (isotropic) treatment of cell e.s.d.'s is used for estimating e.s.d.'s involving l.s. planes.
Refinement. Refinement of *F*^2^ against ALL reflections. The weighted *R*-factor *wR* and goodness of fit *S* are based on *F*^2^, conventional *R*-factors *R* are based on *F*, with *F* set to zero for negative *F*^2^. The threshold expression of *F*^2^ > σ(*F*^2^) is used only for calculating *R*-factors(gt) *etc*. and is not relevant to the choice of reflections for refinement. *R*-factors based on *F*^2^ are statistically about twice as large as those based on *F*, and *R*- factors based on ALL data will be even larger.

### Fractional atomic coordinates and isotropic or equivalent isotropic displacement parameters (Å^2^)

**Table:**

	*x*	*y*	*z*	*U*_iso_*/*U*_eq_	Occ. (<1)
C1	0.27806 (7)	0.38760 (10)	0.16427 (12)	0.0365 (3)	
C2	0.22710 (7)	0.41256 (11)	0.03724 (13)	0.0419 (3)	
H2	0.2498	0.4597	−0.0404	0.05*	
C3	0.14287 (8)	0.36894 (12)	0.02273 (14)	0.0475 (3)	
C4	0.11069 (8)	0.29958 (13)	0.14029 (16)	0.0559 (4)	
H4	0.0542	0.27	0.1339	0.067*	
C5	0.16120 (9)	0.27374 (14)	0.26661 (16)	0.0583 (4)	
H5	0.1383	0.2268	0.3443	0.07*	
C6	0.24561 (8)	0.31628 (12)	0.28041 (13)	0.0478 (3)	
H6	0.2797	0.2975	0.3655	0.057*	
C7	0.42002 (7)	0.44614 (9)	0.28233 (11)	0.0334 (3)	
C8	0.5	0.52114 (14)	0.25	0.0364 (4)	
H8	0.4902 (8)	0.5697 (12)	0.1602 (13)	0.044*	
C9	0.08888 (10)	0.39539 (16)	−0.11743 (19)	0.0696 (5)	
H9A	0.107	0.4699	−0.1608	0.104*	0.5
H9B	0.0286	0.4002	−0.0897	0.104*	0.5
H9C	0.0966	0.333	−0.1913	0.104*	0.5
H9D	0.0478	0.3322	−0.1337	0.104*	0.5
H9E	0.1262	0.4019	−0.2048	0.104*	0.5
H9F	0.0582	0.469	−0.1032	0.104*	0.5
N1	0.36255 (6)	0.43945 (9)	0.16717 (10)	0.0382 (3)	
H1N	0.379	0.4711	0.0825	0.046*	
O1	0.41040 (5)	0.39905 (8)	0.40829 (8)	0.0475 (3)	

### Atomic displacement parameters (Å^2^)

**Table:**

	*U*^11^	*U*^22^	*U*^33^	*U*^12^	*U*^13^	*U*^23^
C1	0.0337 (6)	0.0422 (6)	0.0337 (5)	−0.0052 (4)	−0.0007 (4)	−0.0028 (4)
C2	0.0378 (6)	0.0485 (7)	0.0394 (6)	−0.0059 (5)	−0.0026 (5)	0.0048 (5)
C3	0.0382 (6)	0.0509 (7)	0.0532 (7)	−0.0035 (5)	−0.0085 (5)	−0.0028 (6)
C4	0.0394 (7)	0.0619 (8)	0.0663 (8)	−0.0177 (6)	−0.0014 (6)	−0.0012 (7)
C5	0.0561 (8)	0.0647 (9)	0.0541 (8)	−0.0246 (7)	0.0036 (6)	0.0089 (6)
C6	0.0487 (7)	0.0555 (7)	0.0390 (6)	−0.0129 (6)	−0.0034 (5)	0.0060 (5)
C7	0.0326 (6)	0.0384 (6)	0.0292 (5)	0.0024 (4)	−0.0010 (4)	−0.0029 (4)
C8	0.0322 (8)	0.0390 (8)	0.0380 (8)	0	−0.0048 (6)	0
C9	0.0491 (8)	0.0836 (11)	0.0758 (10)	−0.0062 (7)	−0.0254 (7)	0.0075 (8)
N1	0.0341 (5)	0.0513 (6)	0.0292 (5)	−0.0086 (4)	−0.0016 (4)	0.0051 (4)
O1	0.0478 (5)	0.0633 (6)	0.0313 (4)	−0.0085 (4)	−0.0044 (3)	0.0060 (4)

### Geometric parameters (Å, °)

**Table:**

C1—C2	1.3821 (16)	C7—O1	1.2301 (13)
C1—C6	1.3873 (16)	C7—N1	1.3359 (14)
C1—N1	1.4226 (14)	C7—C8	1.5176 (14)
C2—C3	1.3886 (16)	C8—C7^i^	1.5176 (14)
C2—H2	0.93	C8—H8	0.966 (12)
C3—C4	1.3824 (19)	C9—H9A	0.96
C3—C9	1.5035 (18)	C9—H9B	0.96
C4—C5	1.3754 (19)	C9—H9C	0.96
C4—H4	0.93	C9—H9D	0.96
C5—C6	1.3863 (17)	C9—H9E	0.96
C5—H5	0.93	C9—H9F	0.96
C6—H6	0.93	N1—H1N	0.86
			
C2—C1—C6	119.96 (10)	C3—C9—H9A	109.5
C2—C1—N1	116.31 (10)	C3—C9—H9B	109.5
C6—C1—N1	123.73 (10)	H9A—C9—H9B	109.5
C1—C2—C3	121.62 (11)	C3—C9—H9C	109.5
C1—C2—H2	119.2	H9A—C9—H9C	109.5
C3—C2—H2	119.2	H9B—C9—H9C	109.5
C4—C3—C2	117.93 (11)	C3—C9—H9D	109.5
C4—C3—C9	121.18 (12)	H9A—C9—H9D	141.1
C2—C3—C9	120.89 (12)	H9B—C9—H9D	56.3
C5—C4—C3	120.78 (11)	H9C—C9—H9D	56.3
C5—C4—H4	119.6	C3—C9—H9E	109.5
C3—C4—H4	119.6	H9A—C9—H9E	56.3
C4—C5—C6	121.30 (12)	H9B—C9—H9E	141.1
C4—C5—H5	119.3	H9C—C9—H9E	56.3
C6—C5—H5	119.3	H9D—C9—H9E	109.5
C5—C6—C1	118.40 (11)	C3—C9—H9F	109.5
C5—C6—H6	120.8	H9A—C9—H9F	56.3
C1—C6—H6	120.8	H9B—C9—H9F	56.3
O1—C7—N1	124.49 (10)	H9C—C9—H9F	141.1
O1—C7—C8	120.45 (8)	H9D—C9—H9F	109.5
N1—C7—C8	115.03 (8)	H9E—C9—H9F	109.5
C7—C8—C7^i^	112.59 (13)	C7—N1—C1	129.41 (9)
C7—C8—H8	110.0 (7)	C7—N1—H1N	115.3
C7^i^—C8—H8	106.5 (7)	C1—N1—H1N	115.3
			
C6—C1—C2—C3	0.73 (19)	C2—C1—C6—C5	−1.35 (19)
N1—C1—C2—C3	−178.75 (11)	N1—C1—C6—C5	178.09 (12)
C1—C2—C3—C4	0.32 (19)	O1—C7—C8—C7^i^	78.05 (10)
C1—C2—C3—C9	−179.02 (13)	N1—C7—C8—C7^i^	−103.70 (9)
C2—C3—C4—C5	−0.7 (2)	O1—C7—N1—C1	5.17 (19)
C9—C3—C4—C5	178.61 (15)	C8—C7—N1—C1	−173.01 (11)
C3—C4—C5—C6	0.1 (2)	C2—C1—N1—C7	168.77 (11)
C4—C5—C6—C1	0.9 (2)	C6—C1—N1—C7	−10.69 (19)

Symmetry codes: (i) −*x*+1, *y*, −*z*+1/2.

### Hydrogen-bond geometry (Å, °)

**Table:**

*D*—H···*A*	*D*—H	H···*A*	*D*···*A*	*D*—H···*A*
C6—H6···O1	0.93	2.34	2.9124 (14)	120
N1—H1N···O1^ii^	0.86	2.16	2.9932 (12)	162
C8—H8···O1^ii^	0.97 (1)	2.54 (1)	3.3981 (9)	149 (1)

Symmetry codes: (ii) *x*, −*y*+1, *z*−1/2.
